# Core-Shell Hydrogel Particles Harvest, Concentrate and Preserve Labile Low Abundance Biomarkers

**DOI:** 10.1371/journal.pone.0004763

**Published:** 2009-03-10

**Authors:** Caterina Longo, Alexis Patanarut, Tony George, Barney Bishop, Weidong Zhou, Claudia Fredolini, Mark M. Ross, Virginia Espina, Giovanni Pellacani, Emanuel F. Petricoin, Lance A. Liotta, Alessandra Luchini

**Affiliations:** 1 Department of Dermatology, University of Modena and Reggio Emilia, Modena, Italy; 2 Center for Applied Proteomics and Molecular Medicine, George Mason University, Manassas, Virginia, United States of America; 3 Department of Chemistry and Biochemistry, George Mason University, Manassas, Virginia, United States of America; Tufts University, United States of America

## Abstract

**Background:**

The blood proteome is thought to represent a rich source of biomarkers for early stage disease detection. Nevertheless, three major challenges have hindered biomarker discovery: a) candidate biomarkers exist at extremely low concentrations in blood; b) high abundance resident proteins such as albumin mask the rare biomarkers; c) biomarkers are rapidly degraded by endogenous and exogenous proteinases.

**Methodology and Principal Findings:**

Hydrogel nanoparticles created with a N-isopropylacrylamide based core (365 nm)-shell (167 nm) and functionalized with a charged based bait (acrylic acid) were studied as a technology for addressing all these biomarker discovery problems, in one step, in solution. These harvesting core-shell nanoparticles are designed to simultaneously conduct size exclusion and affinity chromatography in solution. Platelet derived growth factor (PDGF), a clinically relevant, highly labile, and very low abundance biomarker, was chosen as a model. PDGF, spiked in human serum, was completely sequestered from its carrier protein albumin, concentrated, and fully preserved, within minutes by the particles. Particle sequestered PDGF was fully protected from exogenously added tryptic degradation. When the nanoparticles were added to a 1 mL dilute solution of PDGF at non detectable levels (less than 20 picograms per mL) the concentration of the PDGF released from the polymeric matrix of the particles increased within the detection range of ELISA and mass spectrometry. Beyond PDGF, the sequestration and protection from degradation for a series of additional very low abundance and very labile cytokines were verified.

**Conclusions and Significance:**

We envision the application of harvesting core-shell nanoparticles to whole blood for concentration and immediate preservation of low abundance and labile analytes at the time of venipuncture.

## Introduction

The peptidome/metabolome, populated by small circulating proteins, nucleic acids or metabolites, represents a valuable source of biomarker information reflecting the biologic state of the organism [1], [2]. Measurement of circulating biomarker molecules holds great promise as a means to a) detect early stage disease[3], b) stratify patients into distinct risk subgroups, and c) monitor progression or response to therapy [4]. The low-molecular-weight (LMW) region of the blood proteome, which is a mixture of small intact proteins and fragments of large proteins, is an emerging arena for biomarker research [5]. Tissue-derived proteins, that are too large to passively enter the blood stream, can be represented in the circulation as peptides or protein fragments. This LMW region of the proteome is particularly amenable to biomarker discovery based approaches using current mass spectrometry technology.

Nevertheless, despite the recent progress in proteomics discovery and measurement technologies, identification of clinically useful biomarkers has been painfully slow. While this lack of progress is partly due to the inherent analytical difficulties associated with an extraordinarily complex sample matrix such as blood, there are three fundamental and serious physiologic barriers thwarting biomarker discovery and measurement:

1. The foremost problem in biomarker measurement is the extremely low abundance (concentration) of candidate markers in blood, which exist below the detection limits of mass spectrometry and conventional immunoassays. Such a low abundance would be expected for early stage disease since the diseased tissue constitutes a small proportion of the patient's tissue volume. Early-stage disease detection generally provides better overall patient outcomes.
2. The second major problem for biomarker discovery and measurement is the overwhelming abundance of resident proteins such as albumin and immunoglobulins, accounting for 90% of circulating plasma proteins, which confound and mask the isolation of rare biomarkers [6]. In fact, the vast majority of low abundance biomarkers are non-covalently and endogenously associated with carrier proteins, such as albumin, which exist in a billion fold excess compared to the rare biomarker [7].
3. A third serious challenge for biomarker measurement is the propensity for the low abundance biomarkers to be rapidly degraded by endogenous and exogenous proteinases immediately after the blood sample is drawn from the patient. Degradation of candidate biomarkers occurs also during transportation and storage of blood, generating significant false positive and false negative results [8].

The field of nanotechnology offers fresh approaches to address these three fundamental physiologic barriers to biomarker discovery. Recently, we have engineered smart hydrogel core-shell nanoparticles that overcome these three barriers and will do so in one step, in solution [9]. A hydrogel particle is a cross linked particle of sub-micrometer size composed of hydrophilic polymers capable of swelling and contracting as a result of the application of an environmental trigger, e.g., temperature, pH, ionic strength or electric field [10]–[14]. Hydrogel particles have extensive applications in biomedicine and biotechnology [15]–[18] because of their high biocompatibility and unique physiochemical properties.

The nanoparticles simultaneously conduct molecular sieve chromatography and affinity chromatography in one step in solution [9]. The molecules captured and bound within the affinity matrix of the particles are protected from degradation by exogenous or endogenous proteases. Despite the promise of this feasibility study [9], it remained to be proven whether such hydrogel particle technology could be shown to be applicable to a clinically relevant, highly labile, and very low abundance biomarker. To address this challenge we created a new class of core-shell particles and tailored the core bait to specifically capture a model biomarker Platelet Derived Growth Factor (PDGF). In order to study the applicability of hydrogel particles to a real world problem, PDGF was chosen as a highly challenging model for cancer related biomarker analysis because it is present in blood in extremely low concentration (3 ng/mL), with a short half life (2 minutes) [12]. The PDGFs are a family of peptide growth factors that signal through cell surface tyrosine kinase receptors and stimulate various cellular functions including growth, proliferation, and differentiation. Four different polypeptide chains (PDGF-A, -B, -C, and -D) encoded by different genes (chromosomes 4, 7, 11, 22) have been described [19], [20]. PDGF plays a role in angiogenesis and the level of tumor interstitial pressure during tumor progression [21]–[23]. Several new therapeutic agents designed to target PDGF and its receptor are presently in use in the oncology clinic[24]–[28]. Despite this known theranostic value, PDGF can not be measured routinely and accurately in the clinic because of the extreme low abundance and high instability of this low molecular weight growth factor. Beyond PDGF, the sequestration and protection from degradation for a series of additional very low abundance and very labile chemokines such as CCL28, CCL24, and CXCL12 were verified. These chemokines have a concentration in serum of 44 pg/mL [29], 103 pg/mL [30], and 1.5 ng/mL [31], respectively. The half life of chemokines in blood is very short, less tan ten minutes [32]. Chemokines are small cytokines that direct migration of leukocytes, activate inflammatory responses and participate in tumor growth. Chemokines modulate tumor behavior by three important mechanisms: regulation of tumor-associated angiogenesis, activation of a host tumor-specific immunological response, and direct stimulation of tumor cell proliferation in an autocrine fashion. All of these mechanisms are promising drug targets [33].

The incorporation of a bait molecule in the porous latex if hydrogel particles drives the uptake of molecules in solution, shifts the equilibrium towards association with the particles, and assures that captured molecules are preserved from degradation. The bait can be introduced via copolymerization of a monomer carrying the chemical moiety or via loading the chemical moiety with covalent bonding to an already formed hydrogel particle.

**Figure 1:**
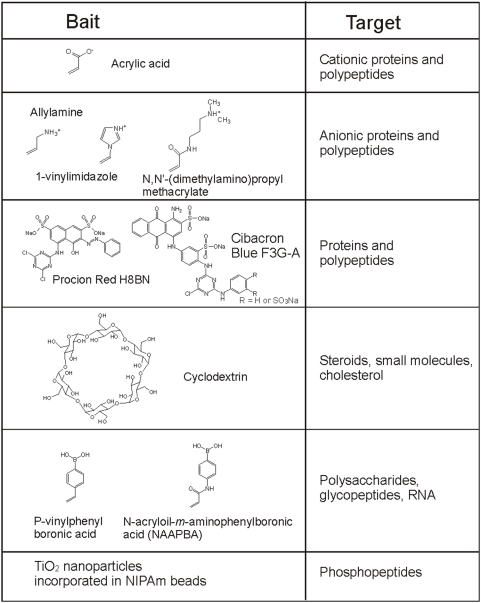
Bait chemistry.

A menu of bait chemistries has been created to selectively bind and concentrate a diverse range of biomarkers, such as a) proteins and peptides, b) metabolites, c) lipids and fatty acids, d) nucleic acids, and e) post translationally modified peptides (e.g. glycosylated and phosphorylated). Bait chemistries include charge-based bait (acrylic acid, allylamine co-monomer), triazine loaded dye (cibacron blue), beta-cyclodextrin, boronic acid. A summary of bait chemistry we are working on is shown in Fig. 1

Acrylic acid is deprotonated at pH values greater than 3.5 and therefore carries a negative charge that targets positively charged polypeptides and proteins. Allylamine (pK = 9.69 [34]) acts as a bait for polypeptides and proteins that have net negative charge. The affinity of polypeptides to charged particles has been proven to be higher than the affinity of polypeptide for carrier proteins and depends on the value of the isoelectric point of proteins and the dissociation constant of particles [9]. Harvesting and concentration properties of charged particles depend on the pH and ionic strength of the solution.

An alternative bait strategy is to load NIPAm based particles with triazine derived textile dyes (Cibacron blue F3G-A, Procion red H8BN) [35]. Dyes have been used in affinity chromatography for their low cost and highly specific molecular recognition [36]. We have successfully synthesized Cibacron Blue dye loaded hydrogel particles and demonstrated their efficacy for uptake small proteins and hormones from urine [37].

Additionally, cyclodextrins were coupled to hydrogel particles. Cyclodextrins are cyclic glucose oligosaccharides that have lipophilic inner cavities and hydrophilic outer surfaces, that are capable of interacting with hydrophobic guest molecules to form noncovalent complexes, and have been extensively used as vector for drug delivery [38]. Cyclodextrin have been shown to bind cholesterol [39], steroids [40], DOPA [41]. Hydrogen bonds, Van der Waals interaction, electrostatic interactions are thought to be the forces that attract and stabilize guest molecules that have suitable size and preferably hydrophobic character [42].

Furthermore, we designed particles that contain boronic acid groups, which are known to form complexes with diol groups of target biomolecules. Boronate ion has been used for affinity chromatography applications involving the selective isolation of nucleotides, RNA, glycated proteins and glycoenzymes [43]–[48].

In a core-shell architecture, the bait containing region is covered by a porous shell. Core-shell hydrogel particles are of special utility because the properties of the core and shell can be tailored separately to suit a particular application. In many core-shell particle systems used for drug delivery, the core is designed to have the properties required for its intended application [12], [49]–[51]. The shell is then separately added to surround and shield the core. The thickness of the shell can be modified to alter permeability or porosity.

**Figure 2:**
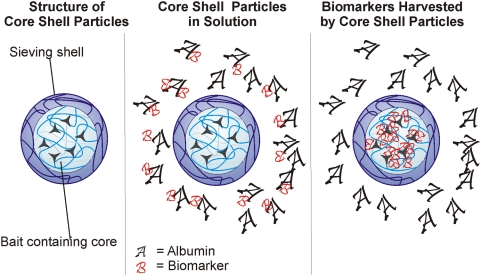
Schematic illustration of core shell particle. The nanoparticle consists of a NIPAm-AAc core that functions as a bait. After adding particles to the protein solution, biomarkers are attracted and entrapped in this bait. A NIPAm shell increases the sieving properties of nanoparticles.

In the present study, we synthesized a core-shell particle in which a *N*-isopropylacrylamide (NIPAm)-acrylic acid (AAc) core contains a charge based bait to perform affinity binding to proteins in solutions, and a NIPAm shell surrounds the core and acts as a sieve to exclude solution phase proteins too large to penetrate the porosity of the shell (Fig. 2). NIPAm based particles exclude larger molecules with a sharp molecular size cut off due to their porous structure. The degree of porosity can be tuned by changing the percentage of cross-linker N,N'methylenebisacrylamide (BIS) with respect to the monomer. At the same time, particles can imbibe large amounts of water which provide favorable conditions for polypeptides and other small molecules to penetrate the polymer matrix, and also allow concentration of rare protein biomarkers [14].

We used three independent experimental systems to test whether the new particles could accomplish the following a) Rapidly harvest all of the solution phase PDGF and chemokine molecules within a complex mixture of high abundance proteins including whole serum, b) Release the captured PDGF and chemokine into a small volume that was a fraction of the starting volume, while completely excluding high abundance proteins such as albumin. This concentration step has the potential to magnify the detectable level of the marker in a small volume that is required for input into a measurement system such as an immunoassay platform or mass spectrometry, and c) Protect the captured PDGF and chemokine from degradation by exogenous degradative enzymes introduced at high concentration. The three independent experimental approaches employed for the present study were 1) A clinical grade ELISA immunoassay, 2) Gel Electrophoresis of the starting solution, the supernatant and the particle contents followed by immunoblotting, and 3) Mass Spectrometry analysis of the starting solution compared to the particle capture eluate.

The purpose of this study was to explore the capacity of the core-shell particles to concentrate and preserve biomarkers as theoretically envisioned.

## Results

### Particle synthesis and characterization

**Figure 3:**
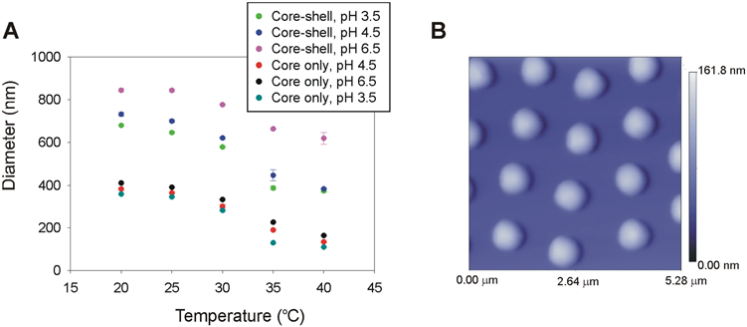
Light scattering and atomic force microscopy characterization of nanoparticles. (A) At room temperature, core is approximately 360 nm in size whereas adding core-shell particles have a diameter of 700 nm at pH 4.5. Core and core shell particles follow a typical temperature dependent behavior. (B) Particle suspension in MilliQ water (pH 5.5, 1 µg/mL) was deposited on freshly cleaved mica under humid atmosphere at room temperature for 15 minutes and dried under nitrogen. Atomic force microscopy (AFM) image of nanoparticles was acquired. Particles have a diameter of approximately 800 nm and exhibit a homogeneous size distribution. The scale bar for particle height shows a maximum height of 168 nm. The AFM picture was acquired under dry status therefore the particles are distorted (flattened) from their spherical shape due to drying on the mica surface.

For the particle architecture used in the present study, a NIPAm shell surrounds a NIPAm/AAc core, containing affinity bait moieties. The sieving capability of the NIPAm shell shields the core and its affinity bait groups from larger molecules that may be present and could compete with the intended low-abundance, low molecular weight molecular targets for binding to the affinity bait in the core. Light scattering characterization was conducted on the particles during synthesis and at the end of the process in order to compare the sizes of the core and the core-shell particles. The core diameter at 25°C and pH 4.5 is 364.7+/−4.3 nm whereas the diameter of the core-shell particles at the same conditions is 699.4+/−6.2 nm (Fig. 3A). This suggests that the thickness of the shell is about 170 nm. Following the characteristic behavior of AAc containing hydrogels, both the core and the core-shell particle size decreased with increasing temperature and decreasing pH (Fig. 3A). Particles were further characterized by means of atomic force microscopy (AFM). AFM particle images (Fig. 3B) confirm size homogeneity, and AFM particle diameter readings were consistent with those measured with light scattering. Particle concentration, as obtained by weighing the lyophilized particles, was 10 mg/mL, and the number of particles per milliliter was 230 million.

### Molecular sieving and concentration of PDGF by core-shell particles

**Figure 4:**
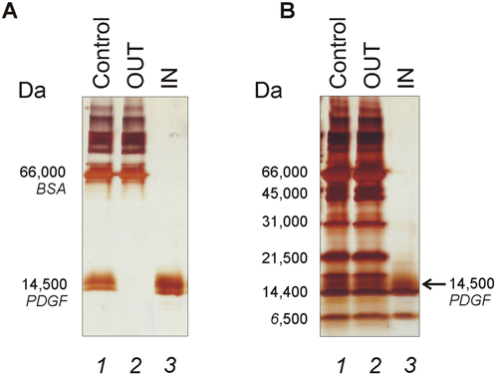
SDS-PAGE Analysis of PDGF incubated particles. (A) Lane 1) Starting solution containing BSA and PDGF (Control), 2) Supernatant (OUT); 3) Particle content (IN). Particles remove PDGF from carrier albumin with a total exclusion of albumin itself. (B) Lane 1) Starting solution containing PDGF, BSA, aprotinin (MW 6,500 Da), lysozyme (MW 14,400 Da), trypsin inhibitor (MW 21,500 Da), carbonic anhydrase (MW 31,000 Da), and ovalbumin (MW 45,000 Da) (Control), 2) Supernatant (OUT); 3) Particle content (IN). Particles harvest PDGF together with low molecular weight proteins and exclude proteins above ca 20,000 Da.

Human platelet derived growth factor (PDGF, MW 14,500 Da) was spiked in a solution containing bovine serum albumin (BSA, MW 66,000 Da) as carrier protein associated with the PDGF. Core-shell hydrogel nanoparticles added to the PDGF-BSA solution acted as a molecular sieve as evidenced by no detectable association of BSA with the particles, and the particles completely sequestered all the solution phase PDGF while completely excluding the BSA (Fig. 4A). This suggests that PDGF affinity for the bait was higher than that for carrier BSA. In order to further assess the molecular sieving properties of core-shell particles, a solution containing: PDGF, BSA, aprotinin (MW 6,500 Da), lysozyme (MW 14,400 Da), trypsin inhibitor (MW 21,500 Da), carbonic anhydrase (MW 31,000 Da), and ovalbumin (MW 45,000 Da), was used. The uptake of proteins by the particles was evaluated by SDS PAGE. Core-shell particles efficaciously captured and concentrated low molecular weight proteins with a weight less than 21,500 Da whereas proteins with high molecular weight remained excluded from the particles (Fig 4B).

### Concentration of PDGF in solution by core-shell hydrogel nanoparticles

We examined the nanoparticles ability to concentrate a dilute PDGF sample, at a concentration below the detection threshold of the ELISA, to determine if the concentration of PDGF could be increased by particle sequestration, rendering the PDGF measurable by the ELISA.

**Figure 5:**
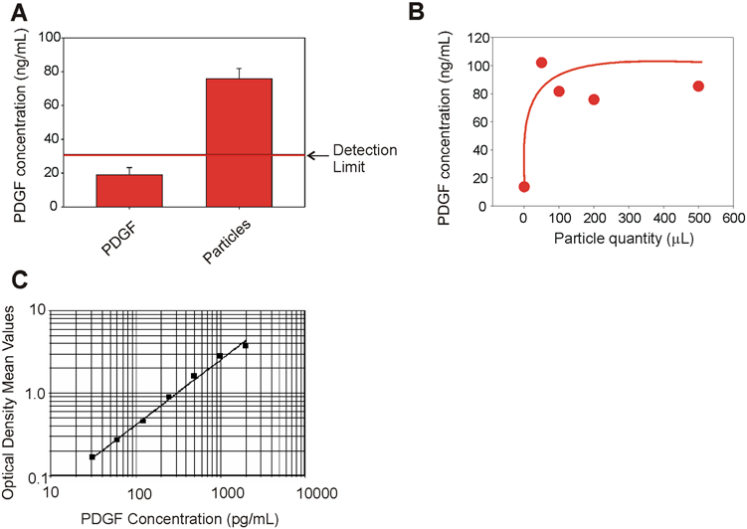
Core shell particles raise the concentration of undetectable PDGF into the detection range of ELISA assay. (A) ELISA readings of the starting solution of PDGF in Calibrator diluent RD6-3 (R&D Systems, animal serum with preservatives) at a concentration of 18.92+/−4.313 pg/mL and PDGF eluted from core-shell particles (85.27+/−2.24 pg/mL). (B) PDGF concentration in the core-shell particle eluate plotted against the quantity of particles utilized for the incubation, duplicate experiments. (C) ELISA standard curve of PDGF concentration versus absorbance. The standard curve was generated with two repeats for each PDGF calibrator concentration.

**Figure 6:**
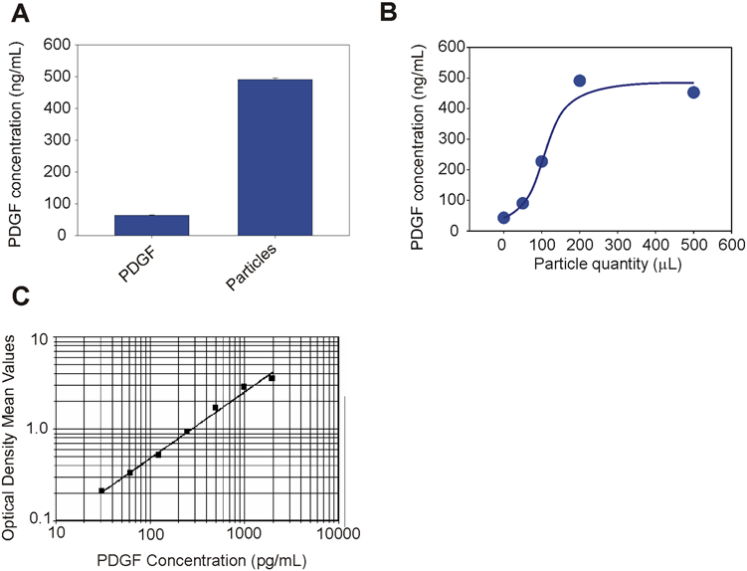
Core shell particles increase the concentration of extremely dilute PDGF approximately 10-folds (1000 percent) as measured by ELISA assay. (A) ELISA readings of the starting solution of PDGF in Calibrator diluent RD6-3 (R&D Systems, animal serum with preservatives) at a concentration of 63.69+/−1.448 pg/mL and PDGF eluted from core-shell particles (491.14+/−4.818 pg/mL). (B) PDGF concentration in core-shell particle eluate plotted against the quantity of particles utilized for the incubation, duplicate experiments. (C) ELISA standard curve of PDGF concentration versus absorbance. The standard curve was generated with two repeats for each PDGF calibrator concentration.

As shown in Fig. 5A, previously undetectable level of PDGF was recovered from the particles and successfully quantified by ELISA at concentrations ranging from 75 to 102 pg/mL. The value of PDGF concentration in the starting solution (18.92+/−4.313 pg/mL) reported in Fig. 5A was below the linear range of the ELISA immunoassay (minimum detectable PDGF dose = 30 pg/mL) and was estimated by using the optical density and extrapolated from the standard curve. Per manufacturer's instructions, the minimum detectable dose was determined by adding two standard deviations to the mean optical density value of twenty zero standard replicates and calculating the corresponding concentration. Therefore, core-shell particles, incubated with a PDGF solution at a concentration undetectable by ELISA, harvested and concentrated PDGF to a level higher than the detection limit of the assay. Saturation was reached with the minimum amount of particles when the PDGF solution was very dilute, as expected (Fig. 5B). A standard curve for PDGF ELISA assay was generated (Fig. 5C) in order to assess the quality of the procedure. A similar experiment was performed with a more concentrated PDGF solution. The concentration of PDGF in the starting solution was 63.69 (+/−1.448) pg/mL whereas the concentration of PDGF recovered from particles was 452.81 (+/−4.818) pg/mL yielding a concentration factor of about 700% (Fig. 6A). A PDGF solution was incubated with different volumes of particles and demonstrated that saturation was reached when the volume of particles was 200 µl (46 million particles, 1∶5 v/v particles∶PDGF solution ratio, Fig. 6B). The standard curve for PDGF ELISA assay was repeated (Fig. 6C).

**Figure 7:**
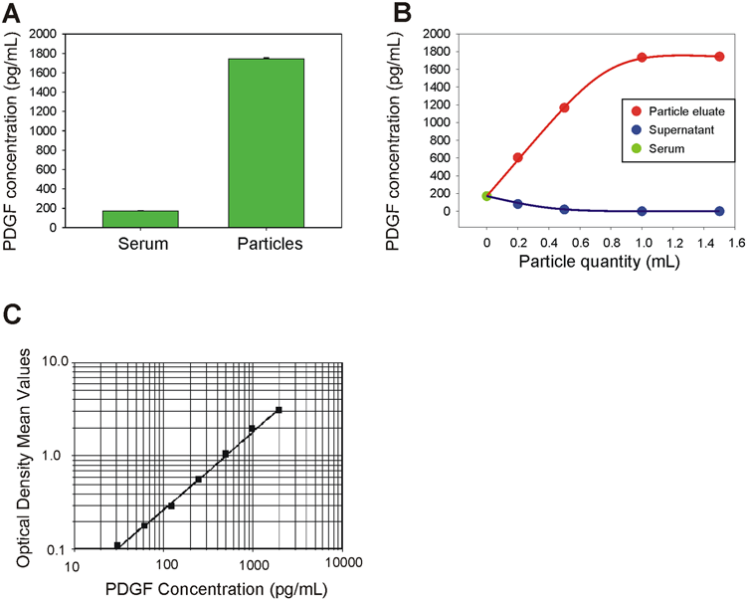
Core shell particles increase the concentration of native PDGF in serum as measured by ELISA assay. (A) ELISA readings of the starting serum solution in Calibrator diluent RD6-3 (R&D Systems, animal serum with preservatives) at a concentration of 170.91+/−4.66 pg/mL and PDGF eluted from core-shell particles (1743.43+/−11.06 pg/mL). (B) PDGF concentration in core-shell particle eluate plotted against the quantity of particles utilized for the incubation, duplicate experiments. (C) ELISA standard curve of PDGF concentration versus absorbance. The standard curve was generated with two repeats for each PDGF calibrator concentration.

A further experiment was performed in order to test the ability of core shell particles to sequester, concentrate and preserve native PDGF from human serum. We examined the effect of excess interfering proteins on the amount of particles necessary to reach saturation and complete depletion of native PDGF from serum. Serum was diluted 1∶10 in Tris HCl 50 mM pH 7 and incubated with increasing quantities of particles (200, 500, 1000, and 1500 µL). The value of PDGF in the starting serum solution was read as 170.92+/−4.66 pg/mL whereas the concentration of PDGF recovered from particles was 1743.43+/−11.06 pg/mL yielding a concentration factor of about 10-fold (1000 percent) (Fig. 7A). Saturation was reached at a value of 1000 µL (230 million particles, 1∶1 v/v particles∶serum solution, Fig. 7B). Given the fact that the starting concentration of PDGF in serum is higher than the concentration of PDGF in the solution of Fig. 6, we can conclude that the presence of serum, with its enormous protein content in the starting sample, requires less than double amount of particles to deplete the sample, thus confirming the extremely high binding capacity of particles, even in the presence of abundant serum proteins. The standard curve for the PDGF ELISA assay used in these studies is shown (Fig. 7C).

### Concentration of chemokines in solution by core-shell particles

**Figure 8:**
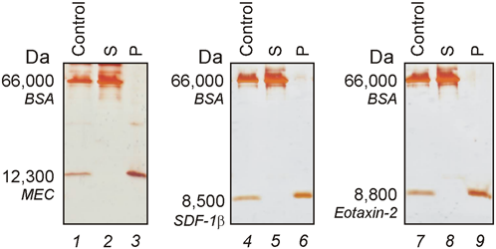
SDS PAGE analysis showing chemokines uptake by particles. Core-shell particles were incubated with the following chemokines, mucosae-associated epithelial chemokine (MEC/CCL28), stromal cell-derived factor-1 beta, (SDF-1β/CXCL12b), and eotaxin-2 (CCL24), in presence of bovine serum albumin (BSA). Solutions of the chemokines and BSA are shown in lanes 1, 4, and 7. After incubation with the particles, no chemokine was left in the supernatant (S, lane 2, 5, and 8) and all the chemokine was captured by particles (P, lanes 3, 6, and 9). BSA was totally excluded by particles.

In Fig. 8 SDS PAGE analysis is shown on core shell acrylic acid functionalized particles incubated with other relevant models for serological biomarker, namely mucosae-associated epithelial chemokine (MEC/CCL28, 12,300 Da), stromal cell-derived factor-1 beta, (SDF-1β/CXCL12b, 8,500 Da), and eotaxin-2 (CCL24, 8,800 Da) mixed with BSA. Chemokines were totally removed from solution, captured and concentrated by particles, whereas BSA was completely excluded.

### Protection from enzymatic degradation of PDGF by core-shell particles

**Figure 9:**
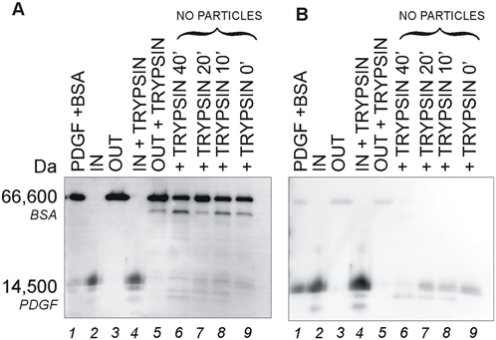
Immunoblot analysis showing that core-shell particles protect captured PDGF from tryptic degradation. (A) Sypro ruby total protein staining and (B) Immunoblot analysis with anti-PDGF antibody of the same PVDF membrane are presented. Lane 1) control PDGF+BSA solution; 2) content of particles incubated with PDGF+BSA (IN); 3) supernatant of particles incubated with PDGF+BSA (OUT); 4) content of particles incubated with BSA+PDGF+trypsin (IN+TRYPSIN); 5) supernatant of particles incubated with BSA+PDGF+trypsin (OUT+TRYPSIN); 6) BSA+PDGF+trypsin without particles incubated for 40 minutes (+TRYPSIN 40′); 7)) BSA+PDGF+trypsin without particles incubated for 20 minutes (+TRYPSIN 20′); 8)) BSA+PDGF+trypsin without particles incubated for 10 minutes (+TRYPSIN 10′); 9)) BSA+PDGF+trypsin without particles incubated for 0 minutes (+TRYPSIN 0′).

Degradation of biomarkers by endogenous and exogenous proteases is a major source of biomarker performance bias, and hinders the discovery and measurement of candidate biomarkers. Immunoblot analysis was used to evaluate the particles ability to protect PDGF from enzymatic degradation. Trypsin action on PDGF in the absence of particles was evident after 10 minutes and almost complete after one hour, as indicated by nearly undetectable PDGF bands at 14,000–17,000 Da (Fig. 9A and Fig. 9B Lanes 6–8). In marked contrast, PDGF incubated with trypsin and core-shell particles generated a single species band that was not diminished in staining intensity and was not fragmented, suggesting that particles successfully preserved PDGF from proteolysis (Fig. 9A and Fig. 9B Lane 4). The PDGF band for the particles loaded with PDGF without trypsin (Fig. 9A and Fig. 9B Lane 2) was identical to that for the particles loaded with PDGF and trypsin (Fig. 9A and Fig. 9B Lane 4) further suggesting that no PDGF protein was lost because of enzymatic degradation.

### Protection from enzymatic degradation of chemokines by core-shell particles

**Figure 10:**
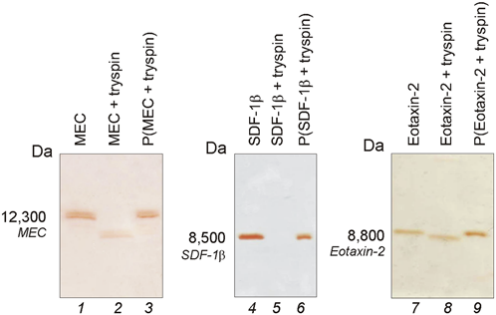
SDS PAGE analysis showing that core-shell particles protect chemokines from enzymatic degradation. Core-shell particles were incubated with the following chemokines, mucosae-associated epithelial chemokine (MEC/CCL28), stromal cell-derived factor-1 beta, (SDF-1β/CXCL12b), and eotaxin-2 (CCL24), in presence of trypsin. Solution of the chemokines (control) are shown in Lanes 1, 4, and 7. Chemokines incubated with particles (Lane 3, 6, and 9) are protected from tryptic degradation whereas chemokines not incubated with particles (Lane 2, 5, and 8) are susceptible to proteolytic digestion.

SDS-PAGE analysis was used to evaluate the particles ability to protect chemokines, chosen as model, from enzymatic degradation. As shown in Fig. 10, trypsin rapidly degraded each class of chemokines in the absence of the sequestration by particles (Fig. 10, Lanes 2, 5, 8). In marked contrast, chemokines incubated with trypsin and particles (Fig. 10, Lane 3, 6, 9) generated a single species band that was not fragmented, suggesting that particles successfully preserved biomarkers from proteolysis.

### Core-shell particles concentrate and preserve PDGF spiked in human serum

The extremely short half-life of PDGF in plasma (2 minutes) is a major analytical challenge. Immunoblotting and mass spectrometry were used to study the efficiency of core-shell particles to harvest, concentrate and preserve PDGF spiked in human serum. Immunoblotting was used to verify the preservation of PDGF by the presence of the correct molecular weight intact protein.

**Figure 11:**
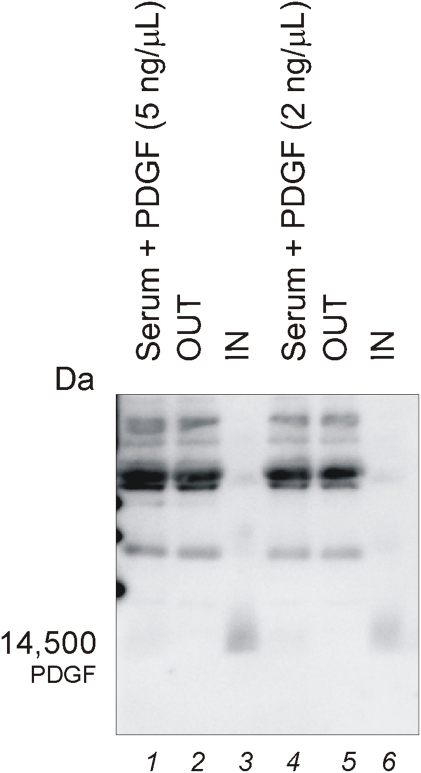
Immunoblot analysis demonstrating recovery of PDGF spiked in human serum. Lane 1) Human serum plus PDGF (5 ng/µL): when serum is not incubated with particles, PDGF cannot be detected; 2) particle supernatant (OUT); 3) particle content (IN); 4) Human serum plus PDGF (2 ng/µL): when serum is not incubated with particles, PDGF cannot be detected; 2) particle supernatant (OUT); 3) particle content (IN).

Aliquots of 50 µL of core-shell particles were incubated with 50 µL of a solution with PDGF (at a concentration of 5 ng/mL or 2 ng/mL) spiked in human serum diluted 1∶25 in 50 mM TrisHCl pH 7 for 1 hour at room temperature. The particles excluded the high molecular weight proteins which remained in the supernatant (Fig. 11, Lane 2 and 5) and at the same time concentrated PDGF (Fig. 11, Lane 3 and Lane 6) that was spiked in serum at two different concentrations. As shown above with the ELISA, PDGF in the starting solution was undetectable (Fig. 11, Lane 1 and 4). The particles increased the concentration of PDGF well above the immunoblot detection limit within an extraordinarily complex serum solution, with no detectable alteration in the mass or abundance of the PDGF molecule.

### Application of core-shell particles to preanalytical sample preparation prior to mass spectrometry analysis of complex protein mixtures

Mass spectrometers have a detection sensitivity two to three orders of magnitude above the concentration of low abundance serum proteins which are expected to provide the most valuable diagnostic information. Mass spectrometry analysis demonstrated that core-shell particles are able to concentrate and preserve PDGF in known protein mixtures and in serum.

As shown in Table 1, mass spectrometry analysis of the solutions containing PDGF at a ratio of 1∶60, 1∶600 and 1∶6,000 to total protein identified 13, 6, and 1 peptides belonging to human PDGF respectively, while eluates from core-shell particles contained 33, 15, 5 peptides respectively. At 1∶60,000 PDGF to total protein ratio, PDGF peptides were not detected in the original solution after trypsin digestion for mass spectrometric analysis (PDGF concentration of 0.67 ng/mL), while eluates from the particles yielded a clear mass spectrometer detection spectra of the PDGF trypsin peptides. Thus core-shell particles increased the number of peptides, identified by mass spectrometry, from a solution when PDGF was present within the known detection level of the mass spectrometer. In addition, when this biomarker was diluted to less than one nanogram per mL and was masked by highly abundant proteins, the core-shell particles were able to concentrate PDGF and raise it into the detection range of mass spectrometry.

**Table 1:** Comparison of identified tryptic peptides from particle elute and starting solution by nano reverse phase liquid chromatography mass spectrometry (RPLC-MS/MS).

Dilution	PDGF concentration (ng/ml)	Identified tryptic peptides from Particle Elute a	Peptide Ions b	Peptide Hits c	Identified tryptic peptides from Starting Solution a	Peptide Ions b	Peptide Hits^c^
1∶60	667	K.KATVTLEDHLACK.C	18/24	33	K.KATVTLEDHLACK.C	17/24	13
		K.KATVTLEDHLACK.C	18/24		K.KATVTLEDHLACK.C	18/24	
		K.ATVTLEDHLACK.C	16/22		K.ATVTLEDHLACK.C	16/22	
		K.KATVTLEDHLACK.C	19/24		K.TRTEVFEISR.R	16/18	
		K.KATVTLEDHLACK.C	27/48		K.TRTEVFEISR.R	15/18	
		K.KATVTLEDHLACK.C	18/24		K.ATVTLEDHLACK.C	17/22	
		K.TRTEVFEISRR.L	13/20		K.TRTEVFEISR.R	16/18	
		K.ATVTLEDHLACK.C	16/22		K.TRTEVFEISR.R	22/36	
		K.TRTEVFEISR.R	17/18		R.TEVFEISR.R	13/14	
		K.ATVTLEDHLACK.C	17/22		R.SLGSLTIAEPAMIAECK.T	21/32	
		K.ATVTLEDHLACK.C	15/22		R.TNANFLVWPPCVEVQR.C	19/30	
		K.TRTEVFEISR.R	15/18		R.TNANFLVWPPCVEVQR.CR.TNANFLVWPPCVEVQR.C	22/30	
		K.ATVTLEDHLACK.C	17/22			22/30	
		R.NVQCRPTQVQLRPVQVR.K	26/64				
		K.TRTEVFEISR.R	16/18				
		K.TRTEVFEISR.R	23/36				
		K.ATVTLEDHLACK.C	14/22				
		K.TRTEVFEISR.R	17/18				
		K.ATVTLEDHLACKCETVAAAR.P	34/76				
		R.TEVFEISR.R	13/14				
		R.SLGSLTIAEPAMIAECK.T	15/32				
		R.SLGSLTIAEPAMIAECK.T	21/32				
		R.SLGSLTIAEPAMIAECK.T	41/64				
		R.TNANFLVWPPCVEVQR.C	18/30				
		R.TNANFLVWPPCVEVQR.C	22/30				
		R.LIDRTNANFLVWPPCVEVQR.C	32/76				
		R.TNANFLVWPPCVEVQR.C	23/30				
		R.TNANFLVWPPCVEVQR.C	23/30				
		R.TNANFLVWPPCVEVQR.C	17/30				
		R.TNANFLVWPPCVEVQR.C	13/30				
		R.TNANFLVWPPCVEVQR.C	15/30				
		R.TNANFLVWPPCVEVQR.C	14/30				
		R.TNANFLVWPPCVEVQR.C	16/30				
1∶600	66.7	K.KATVTLEDHLACK.C	19/24	15	K.KATVTLEDHLACK.C	18/24	6
		K.ATVTLEDHLACK.C	13/22		R.NVQCRPTQVQLRPVQVR.K	26/64	
		K.KATVTLEDHLACK.C	19/24		K.TRTEVFEISR.R	15/18	
		K.ATVTLEDHLACK.C	16/22		R.TEVFEISR.R	13/14	
		K.ATVTLEDHLACK.C	14/22		R.TNANFLVWPPCVEVQR.CR.TNANFLVWPPCVEVQR.C	16/30	
		K.TRTEVFEISR.R	16/18			20/30	
		K.ATVTLEDHLACK.C	16/22				
		R.NVQCRPTQVQLRPVQVR.K	28/64				
		K.TRTEVFEISR.R	15/18				
		R.TEVFEISR.R	13/14				
		R.SLGSLTIAEPAMIAECK.T	21/32				
		R.TNANFLVWPPCVEVQR.C	19/30				
		R.TNANFLVWPPCVEVQR.C	19/30				
		R.TNANFLVWPPCVEVQR.C	21/30				
		R.TNANFLVWPPCVEVQR.C	21/30				
1∶6,000	6.67	K.ATVTLEDHLACK.C	16/22	5	R.NVQCRPTQVQLRPVQVR.K	24/64	1
		R.NVQCRPTQVQLRPVQVR.K	27/64				
		K.TRTEVFEISR.R	15/18				
		R.TNANFLVWPPCVEVQR.C	14/30				
		R.TNANFLVWPPCVEVQR.C	20/30				
1∶60,000	0.667	K.ATVTLEDHLACK.C	13/22	1	PDGF was not detected		0

a The amino acids between the dots are the identified tryptic peptide sequence, and the amino acid before or after the dot is the upstream or downstream residue of this peptide.

b Peptide Ions is the ratio of number of matched b-ions and y-ions to the number of theoretical b-ions and y-ions for the identified peptide based on Sequest database search result.

c Peptide Hits is the total number of identified peptides (including the same peptide with the same charge, the same peptide with different charge, and the different peptide) from one protein based on Sequest database search result.

In order to further assess the capability of particles to concentrate and preserve PDGF in a physiologic medium, and to identify any proteins sequestered simultaneously, PDGF (at a concentration of 5 ng/µL) was spiked in human serum diluted 1∶25 in 50 mM TrisHCl pH 7 and the solution was incubated with 50 µl of core-shell particles for 1 hour. Proteins were eluted, dried and analyzed with nanoRPLC-MS/MS. As shown in Table S1, PDGF was recovered and clearly identified with high peptide hit coverage by mass spectrometer analysis. A number of rare and low molecular weight proteins were identified, within the mixture of proteins captured by the core-shell particles. Additionally, core-shell particles were incubated with human serum and the proteins eluted from the particles were analyzed with nanoRPLC-MS/MS. Peptides belonging to native PDGF present in blood at very low abundance (∼1 ng/mL) were identified. Examples of identified rare proteins are reported in Table 2.

**Table 2:** Native PDGF was found in healthy donor serum purified by core shell particles and measured by nano reverse phase liquid chromatography mass spectrometry (RPLC-MS/MS).

Reference	Accession^a^	P_pep_ b	S_f_ c	Score^d^	MW^e^	ePeptide
properdin P factor, complement	4505737	2.22E-15	4.56	50.27	51242.0	6
vinculin isoform meta-VCL	7669550	5.55E-15	11.49	130.23	123721.9	13
talin 1	16753233	1.22E-14	32.50	350.29	269497.3	38
angiogenin, ribonuclease, RNase A family, 5 precursor	4557313	2.70E-13	6.45	70.24	16539.4	10
serum amyloid A4, constitutive	10835095	4.89E-12	2.89	30.26	14797.3	4
serum deprivation response protein	4759082	3.23E-10	1.85	20.22	47144.6	2
peroxiredoxin 6	4758638	4.14E-09	0.90	10.20	25019.2	1
glyceraldehyde-3-phosphate dehydrogenase	7669492	2.26E-08	1.66	20.19	36030.4	2
tissue inhibitor of metalloproteinase 3 precursor	4507513	2.57E-08	1.88	20.17	24128.8	2
parvin, beta isoform b	20127528	4.09E-08	0.96	10.17	41688.1	1
chemokine (C-C motif) ligand 28	22538811	7.08E-08	0.98	10.22	14270.4	1
talin 2	22035665	8.11E-08	1.52	20.16	271382.8	2
S100 calcium-binding protein A9	4506773	1.05E-07	0.95	10.20	13233.5	1
ras suppressor protein 1 isoform 2	34577083	2.56E-07	1.76	20.25	25529.6	2
serum amyloid P component precursor	4502133	3.33E-07	1.86	20.15	25371.1	2
tubulin, alpha, ubiquitous	57013276	4.45E-07	2.67	30.17	50119.6	3
chemokine (C-X-C motif) ligand 12	76563933	6.29E-07	2.73	30.21	13696.7	3
enolase 1	4503571	7.04E-07	0.80	10.15	47139.4	1
cathelicidin antimicrobial peptide	39753970	1.08E-06	0.90	10.13	19289.2	1
cell division cycle 42 isoform 1	89903012	1.37E-06	0.94	10.16	21245.0	1
cardiac muscle alpha actin proprotein	4885049	1.59E-06	4.25	50.21	41991.9	5
PREDICTED: similar to ARP3 actin-related protein 3 homolog B	113419329	1.79E-06	0.95	10.15	43634.8	1
brain-derived neurotrophic factor isoform a preproprotein	25306253	2.20E-06	1.87	20.19	27800.0	2
brain-derived neurotrophic factor isoform c preproprotein	25306261	2.20E-06	1.87	20.19	29798.0	2
PREDICTED: similar to Prostate, ovary, testis expressed protein on chromosome 2	113413194	4.56E-06	3.09	40.17	121366.6	4
defensin, alpha 1 preproprotein	4758146	5.92E-06	0.91	10.15	10194.2	2
defensin, alpha 3 preproprotein	4885179	5.92E-06	0.91	10.15	10238.2	2
small inducible cytokine A24	22165427	6.03E-06	0.93	10.14	13124.8	1
villin 2	21614499	9.13E-06	0.92	10.14	69369.8	1
radixin	4506467	9.13E-06	0.92	10.14	68521.5	1
**platelet-derived growth factor beta isoform 1, preproprotein**	**4505681**	**1.75E-05**	**0.91**	**10.15**	**27266.1**	**1**
**platelet-derived growth factor beta isoform 2, preproprotein**	**15451786**	**1.75E-05**	**0.91**	**10.15**	**25486.2**	**1**
kininogen 1	4504893	1.81E-05	1.77	20.19	47852.7	2
S100 calcium-binding protein A8	21614544	2.03E-05	0.93	10.16	10827.7	1
transgelin 2	4507357	1.06E-04	0.88	10.14	22377.2	1
ras-related GTP-binding protein	33695095	1.50E-04	0.93	10.13	22454.6	1
vitamin D-binding protein precursor	32483410	6.18E-04	0.87	10.18	52883.0	1

a “Accession” displays the unique protein identification number for the sequence.

b “P_pep_” displays the probability value for the peptide.

c “S_f_” displays the final score that indicates how good the protein match is.

d “score” displays a value that is based upon the probability that the peptide is a random match to the spectral data.

e “Peptide” displays the total number peptide matches.

These data support the use of the core-shell particles to harvest and preserve known biomarkers from serum, while providing a means to dramatically increase the concentration of biomarkers and the effective sensitivity of current biomarker measurement and discovery technology.

## Discussion

(NIPAm/AAc)core–(NIPAm)shell hydrogel particles have been synthesized and successfully applied to harvest, concentrate, and protect from degradation, PDGF and various chemokines, low abundance labile clinical biomarkers from model solutions and serum, a known complex biologic fluid targeted for biomarker discovery. These functions were studied using three independent experimental systems: a quantitative highly sensitive immunoassay, immunoblotting, and mass spectrometry. PDGF and chemokines, chosen as model clinical biomarker analytes, was completely separated from carrier albumin, concentrated, and fully preserved, within minutes, when spiked in model solutions and in human serum. Particle sequestered PDGF and chemokines were fully protected from exogenously added tryptic degradation. Starting with a dilute non detectable concentration of PDGF in the starting solution, the particles partially purified and concentrated this analyte by several orders of magnitude (depending on the starting volume versus the elution volume ratio) into a smaller volume, thereby bringing it into the detection range of a clinical grade PDGF ELISA. Hydrogel core-shell particles harvested and concentrated dilute PDGF along with other low abundance proteins from a complex mixture of high abundance proteins to constitute a new preanalytical method for mass spectrometer based biomarker discovery. The concentration step afforded by the particles appeared to significantly increase the yield of detectable mass spectrometry peptides greater than ten fold compared to direct analysis of the starting solution by conventional sample prep methods.

A major source of biomarker measurement bias and variability is the degradation of biomarkers within the blood sample immediately after venipuncture and during shipment and storage. We envision the application of harvesting core-shell nanoparticles to whole blood for concentration and immediate preservation of low abundance and labile analytes at the time of venipuncture. Accordingly we have tested the use of lyophilized nanoparticles pre-loaded into blood collection vacutainers. Because of the relative small size and mass of the particles, whole blood containing nanoparticles can be centrifuged to remove the blood cells while leaving the nanoparticles in solution for subsequent collection and elution of their contents.

Hydrogel based core-shell particles can be produced in large quantities at low cost, are very reproducible and very uniform in size (diameter∼700 nanometers); particles are stable at room temperature indefinitely.

Nanoparticle harvesting has applications to the proteomic analysis of body fluids beyond blood, such as urine [37], cerebrospinal fluid, and amniotic fluid, which are promising biologic sources for biomarker discovery and measurements [52], [53]. We have created a variety of core baits which have affinity for proteins, peptides, phosphoproteins, glycoprotein's, metabolites, and nucleic acids. Thus it is realistic to propose the use of a mixture of nanoparticles containing subpopulations specifically designed to target a separate class of analyte. In this way, harvesting nanoparticles can be applied to the multiplex analysis of a panel of analytes within a single fluid sample.

## Materials and Methods

The serum used in this study was obtained under an IRB approved serum collection protocol (protocol number GMU HSRB #6081) under informed consent and the data were analyzed anonymously in compliance with HIPAA and the principles expressed in the Declaration of Helsinki.

### Synthesis of core-shell hydrogel particles

Particles were synthesized using NIPAm (Sigma-Aldrich) and BIS (Sigma-Aldrich) by precipitation polymerization [49]. AAc (Sigma-Aldrich) was incorporated into NIPAm particle to provide a charge based affinity moiety bait for affinity capture of peptides and small molecules [9].

### (NIPAm/AAc) core

NIPAm (0.184 g), BIS (0.0055 g), and AAc (48.4 µL) were dissolved in 30 mL of H_2_O and then passed through a 0.2 µm filter. The solution was purged with nitrogen for 15 min at room temperature and medium stir rate and then heated to 70°C. Ammonium persulfate (APS, Sigma-Aldrich, 0.0099 g) in 1 mL of H_2_O was added to the solution to initiate polymerization. After 10 minutes shell solution was added.

### (NIPAm)shell

The shell solution was prepared by dissolving 0.736 g of NIPAm and 0.120 g of BIS in 10 ml of water. The solution was passed through a 0.2 µm filter and purged with nitrogen for 15 min at room temperature and medium stir rate. After 10 minutes from APS injection, shell solution was added to the reacting core solution. The reaction was maintained at 70°C under nitrogen for 3 h and then cooled overnight. Particles were washed to eliminate un-reacted monomer by subsequent centrifugations at 16.1 rcf, 25°C, 15 minutes. Supernatant was disposed and particles re- suspended in 1 ml of water.

### Characterization of Particles

The concentration of particles was assessed by weighing the lyophilized particles. Particles were counted by flow cytometry.

Particle size dependence on temperature and pH was determined via photon correlation spectroscopy (submicron particle size analyzer, Beckman Coulter). The pH of solution was controlled by adding proper amounts of NaOH, HCl with background electrolyte solution of KCl. Average values were calculated for three measurements using a 200 s integration time, and the solutions were allowed to thermally equilibrate for 10 min before each set of measurements. Measured values were then converted to particle sizes via the Stokes-Einstein relationship [54]. Particles were further characterized by atomic force microscopy (AFM) using an NSCRIPTOR™ DPN® System (NanoInk). Particle suspension in MilliQ water (pH 5.5, 1 µg/mL) was deposited on freshly cleaved mica under humid atmosphere at room temperature for 15 minutes and dried under nitrogen before measurement. Images were acquired under AC mode using a silicon tip with a typical resonance frequency of 300 kHz and a radius smaller than 10 nm.

### Particle incubation

50 µL of core-shell particles were incubated with 50 µL of solution containing:

- 0.02 mg/mL PDGF, 0.2 mg/mL BSA in 50 mM TrisHCl pH 7;
- PDGF, BSA, aprotinin (MW 6,500 Da), lysozyme (MW 14,400 Da), trypsin inhibitor (MW 21,500 Da), carbonic anhydrase (MW 31,000 Da), and ovalbumin (MW 45,000 Da), each at a concentration of 0.05 mg/mL dissolved in 50 mM Tris pH 7.
- mucosae-associated epithelial chemokine (MEC/CCL28, Antigenix America), stromal cell-derived factor-1 beta, (SDF-1β/CXCL12b, Antigenix America), and eotaxin-2 (CCL24, Antigenix America) each at a concentration of 0.02 mg/mL mixed with BSA (0.2 mg/mL) and dissolved in 50 mM Tris pH 7.

Incubations lasted 30 minutes at room temperature. After incubation, samples were centrifuged for 7 minutes, 25°C at 16,1 rcf and supernatant was saved. Then, the particles were re-suspended in 1 mL water and centrifuged for 7 minutes, 25°C at 16,1 rcf. Centrifugation and washing were repeated three times.

### Particle elution of captured analytes

The particles were directly loaded on the gel when performing SDS-PAGE or immunoblot analysis. When performing ELISA and mass spectrometry analysis washed particles were incubated with elution buffer (60% acetonitrile-2% acetic acid) for 30 minutes and then centrifuged for 7 minutes, 25°C at 16,1 rcf. Eluate was saved, a second elution step was performed and the eluate saved in the same vial. Samples were then dried with Speed Vac (ThermoFisher) and analyzed with ELISA or mass spectrometry.

### SDS-PAGE analysis

Particles and supernatant deriving from particle incubation were loaded on 18% Tris Glycine gel (Invitrogen Corporation). The particles were retained in the stacking region of the gel while all of the captured proteins were electroeluted from particles and resolved in the gel. Proteins were detected by silver staining.

### Enzymatic Degradation analysis

PDGF-BSA (Cell Signaling Technology) solution (0.11 mg/mL total protein) in 50 mM TrisHCl pH 7 was incubated with trypsin (Promega Corporation) at 1∶100 w/w protein∶protease ratio for different time periods (0, 10, 20, and 40 minutes) at 37°C in order to study the degradation patterns over time. Core-shell particles were incubated for 1 hour at 37°C in a 50 mM TrisHCl pH 7 solution containing PDGF-BSA (0.11 mg/mL total protein) and trypsin (0.0011 mg/mL).

Each of the following chemokines MEC/CCL28, SDF-1β/CXCL12b, CCL24 dissolved in 50 mM TrisHCl pH 7 at a concentration of 0.02 mg/mL was incubated separately with trypsin at 1∶50 w/w protein∶protease ratio and with core-shell particles for 40 minutes at 37°C.

### Immunoblot analysis

Proteins were separated by 1-D gel electrophoresis in 18% Tris-Glycine gel as before, and then transferred onto Immobilion PVDF membrane (Millipore). The membrane was stained with SYPRO Ruby stain (MolecularProbes) according to vendor instructions. The protein blot was imaged using Kodak 4000 MM. The membrane was then incubated with PBS supplemented with 0.2% I-Block (Applied Biosystems/Tropix) and 0.1% Tween 20 (Sigma-Aldrich) for 1 hour at room temperature, and then with antibody raised against PDGF-BB overnight at 4°C under continuous agitation. After washes with PBS supplemented with 0.2% I-Block (w/v) and 0.1% Tween 20, immunoreactivity was revealed by using a specific horseradish peroxidase conjugated anti-IgG secondary antibody and the enhanced chemiluminescence system (Supersignal West Dura, ThermoFisher Scientific).

### ELISA analysis

Particles were incubated with 1 mL of PDGF-BB standard (R&D System) diluted in Calibrator Diluent RD6-3. Incubation times, washings, and elutions were carried out as previously described. Eluate dried with Speed Vac was re-suspended in 100 µL of water with gentle vortexing and then an ELISA assay for Human PDGF-BB was performed according to manufacturer's instructions. Each measurement was carried out in duplicate and individual standard curves were generated for each set of samples assayed. Aliquots of 1 mL of PDGF solution below the detection limit of the kit (20 pg/mL) were incubated for 30 minutes with different numbers of particles (50, 100, 200, and 500 µl). Proteins were eluted from the washed particles by means of two subsequent elution steps with 60% acetonitrile and 2% acetic acid. ELISA readings were performed on a volume of 100 µL.

ELISA measurement of native serum PDGF was used to judge the particle capture yield for a series of particle concentrations introduced in serum. Aliquots of 1000 µL of serum diluted 1∶10 in 50 mM Tris HCl pH were incubated with increasing quantities of particles (200, 500, 1000, and 1500 µL) for 30 minutes at room temperature. Particles were washed as previously described and incubated with 100 µL of elution buffer (60% acetonitrile-2% acetic acid) for 10 minutes and then centrifuged (7 minutes, 25°C at 16.1 rcf). Particle eluates were freeze dried and resuspended in Calibrator Diluent RD6-3, R&D Systems. Serum solution was diluted in Calibrator Diluent. ELISA readings were performed on a volume of 100 µL.

### Mass spectrometry analysis

The following solutions were incubated with the core-shell particles:

- *PDGF spiked in complex protein mixture:* a protein mixture containing 6.7 µg/mL BSA (ThermoFisher Scientific, MW 66,000), 6.7 µg/mL aprotinin (Sigma-Aldrich, MW 6,500 Da), 6.7 µg/mL lysozyme (Sigma-Aldrich, MW 14,400 Da), 6.7 µg/mL trypsin inhibitor (Invitrogen Corporation, MW 21,500 Da), 6.7 µg/mL carbonic anhydrase (Sigma-Aldrich, MW 31,000 Da), and 6.7 µg/mL ovalbumin (Sigma-Aldrich, MW 45,000 Da) in 50 mM TrisHCl pH 7 with a total protein concentration of 40 µg/mL was used. PDGF was added at the following concentrations: 670 ng/mL, 67 ng/mL, 6.7 ng/mL, 0.67 ng/mL so that the ratio between PDGF and the total protein was 1∶60, 1∶600, 1∶6,000 and 1∶60,000 respectively. Aliquots of 1.5 mL of solution were incubated with 100 µL of core-shell particles. Proteins were eluted with 60% acetonitrile and 2% acetic acid in a volume of 100 µL, eluates were dried with a Speed Vac (Thermo) and analyzed with nano reverse phase liquid chromatography mass spectrometry (RPLC-MS/MS). Aliquots of 100 µL were analyzed both from solution and eluates, thus maintaining the same volume.
- *human serum:* 200 µL of healthy donor serum was diluted 1∶3 in TrisHCl buffer, pH 7 50 mM, with 100 µL of core-shell particles. Particles were washed with 10% acetonitrile, 0.5×PBS buffer, and eluted with acetonitrile 60%, acetic acid 2%. Sample was dried with Speed Vac (Thermo) and analysed with nanoRPLC-MS/MS.

Eluates from the particles were analyzed with nanoRPLC-MS/MS. Proteins dried with Speed Vac were reconstituted in 8 M urea, reduced by 10 mM DTT, alkylated by 50 mM iodoacetamide, and digested by trypsin at 37°C overnight. Tryptic peptides were further purified by Zip-Tip (Millipore) and analyzed by LC-MS/MS using a linear ion-trap mass spectrometer (LTQ, Orbitrap). After sample injection, the column was washed for 5 minutes with mobile phase A (0.4% acetic acid) and peptides eluted using a linear gradient of 0% mobile phase B (0.4% acetic acid, 80% acetonitrile) to 50% mobile phase B in 30 minutes at 250 nanoliter/min, then to 100% mobile phase B for an additional 5 minutes. The LTQ mass spectrometer was operated in a data-dependent mode in which each full MS scan was followed by five MS/MS scans where the five most abundant molecular ions were dynamically selected for collision-induced dissociation (CID) using a normalized collision energy of 35%. Tandem mass spectra were searched against SEQUEST database using tryptic cleavage constraints. High-confidence peptide identifications were obtained by applying the following filters to the search results: cross-correlation score (XCorr)> = 1.9 for 1+, 2.2 for 2+, 3.5 for 3+, and a maximum probability for a random identification of 0.01.

## Supporting Information

Table S1 Concentration of PDGF spiked in human serum by core-shell particles. Mass spectrometry analysis of proteins eluted from core shell particles incubated with human serum containing PDGF spiked at a concentration of 5 ng/mL.
(0.03 MB XLS)
